# Arrestin beta 1 Regulates Alveolar Progenitor Renewal and Lung Fibrosis

**DOI:** 10.35534/jrbtm.2024.10006

**Published:** 2024-04-30

**Authors:** Guanling Huang, Yan Geng, Vrishika Kulur, Ningshan Liu, Xue Liu, Forough Taghavifar, Jiurong Liang, Paul W. Noble, Dianhua Jiang

**Affiliations:** 1 Division of Pulmonary, Women’s Guild Lung Institute, Department of Medicine, Cedars-Sinai Medical Center, Los Angeles, CA 90048, USA;; 2 Current Address: GH, Sanofi, 500 Kendall Street, Cambridge, MA 02142, USA; 3 Current Address: YG, Jiangnan University, 1800 Lihu Avenue, Wuxi, Jiangsu, 214122, China; 4 Department of Biomedical Sciences, Cedars-Sinai Medical Center, Los Angeles, CA 90048, USA

**Keywords:** Lung, Fibrosis, IPF, Arrb1, AEC2, Alveolar stem cell, CCL7, Stem cell

## Abstract

The molecular mechanisms that regulate progressive pulmonary fibrosis remain poorly understood. Type 2 alveolar epithelial cells (AEC2s) function as adult stem cells in the lung. We previously showed that there is a loss of AEC2s and a failure of AEC2 renewal in the lungs of idiopathic pulmonary fibrosis (IPF) patients. We also reported that beta-arrestins are the key regulators of fibroblast invasion, and beta-arrestin 1 and 2 deficient mice exhibit decreased mortality, decreased matrix deposition, and increased lung function in bleomycin-induced lung fibrosis. However, the role of beta-arrestins in AEC2 regeneration is unclear. In this study, we investigated the role and mechanism of Arrestin beta 1 (ARRB1) in AEC2 renewal and in lung fibrosis. We used conventional deletion as well as cell type-specific deletion of *ARRB1* in mice and found that *Arrb1* deficiency in fibroblasts protects mice from lung fibrosis, and the knockout mice exhibit enhanced AEC2 regeneration in vivo, suggesting a role of fibroblast-derived ARRB1 in AEC2 renewal. We further found that *Arrb1*-deficient fibroblasts promotes AEC2 renewal in 3D organoid assays. Mechanistically, we found that CCL7 is among the top downregulated cytokines in *Arrb1* deficient fibroblasts and CCL7 inhibits AEC2 regeneration in 3D organoid experiments. Therefore, fibroblast ARRB1 mediates AEC2 renewal, possibly by releasing chemokine CCL7, leading to fibrosis in the lung.

## 1. Introduction

The molecular mechanisms that regulate progressive tissue fibrosis remain poorly understood. Although the causes of pulmonary fibrosis remain largely unknown, it is believed that progressive pulmonary fibrosis is an epithelial-fibroblastic disorder that results from numerous microinjuries to the alveolar epithelia that lead to excessive fibroblast activation, deposition of extracellular matrix, and eventually loss of normal lung architecture [1]. Lung fibrosis is regulated by a variety of processes including TGFbeta signaling [2,3], metabolism [4–6], non-coding RNAs [7–9], epigenetic changes [10], as well as aging [11].

In the lung, type 2 alveolar epithelial cells (AEC2s) function as adult stem cells [12]. Our previous work showed that there is a loss of AEC2s [13,14] and a failure of AEC2 renewal in the lungs of idiopathic pulmonary fibrosis (IPF) patients [13]. We and others have showed that AEC2 renewal is regulated by several pathways [13,15–19] and metabolic reprogramming [6,20]. Furthermore, AEC2 stem cell activity is also regulated by the AEC2 niche [18,21–23], including basal-like cells [24], fibroblasts [18,25,26], endothelial cells [20,27,28], macrophages [17,29], and T cells [30], through various mechanisms such as extracellular vesicle trafficking [18], cytokine release [18,24], or cell–cell interactions.

Beta-arrestins are classically known to participate in agonist-mediated G-protein-coupled receptor (GPCR) signaling through receptor desensitization and internalization [31–33], playing a wide range of roles in development, cancer, asthma [34,35], and tissue fibrosis [36]. We previously reported that beta-arrestin 1 and 2 deficient mice exhibit decreased mortality, decreased matrix deposition, and increased lung function in a model of bleomycin-induced lung fibrosis [37], and serve as the key regulators of fibroblast invasion [37]. However, the role of beta-arrestins in AEC2 regeneration is unclear.

In this study, we investigated the role of Arrestin beta 1 (ARRB1) [38] in AEC2 renewal and in lung fibrosis. We investigated the role of ARRB1 in regulating AEC2 regeneration using conventional deletion as well as cell type-specific deletion of ARRB1 in mice with a lung fibrosis mouse model. We found that targeted deletion of ARRB1 in fibroblasts promoted AEC2 regeneration and ameliorated lung fibrosis.

## 2. Materials and Methods

### 2.1. Mice

*Arrb1*^−/−^ mice (Strain #: 011131, full name B6.129X1(Cg)-*Arrb1*^*tm1Jse*^/J) [39] and wild type (WT) C57BL/6J were purchased from The Jackson Laboratory. *Arrb1*^−/−^ mice were crossbred onto C57BL/6J background for more than five generations. *Arrb1*^flox/flox^ mice [37] were from R. Lefkowitz of Duke University. Col1a2-Cre mice were from Dr. P. Angel of German Cancer Research Center (Das Deutsche Krebsforschungszentrum, DKFZ) [40]. SPC-Cre mice were described previously [41]. All mice were housed in a pathogen-free facility at Cedars-Sinai Medical Center. All animal experiments were approved by the Institutional Animal Care and Use Committee at Cedars-Sinai Medical Center (IACUC008529).

### 2.2. Bleomycin-induce Lung Injury and Fibrosis in Mice

Bleomycin-induce lung injury and fibrosis in age matched 8- to 12-week mice was described previously [13]. Under anesthesia, the trachea was surgically exposed. 1.25 U/kg bleomycin (Hospira, Lake Forest, IL, USA) in 25 mL PBS was instilled into the mouse trachea with a 25-G needle inserted between the cartilaginous rings of the trachea. On day 0, day 7, and day 14, mice were sacrificed and lung tissues were collected for experiments.

### 2.3. Mouse Lung Tissue Digestion

Mouse lung tissue digestion was described previously [6,12,13,42]. In brief, mouse lungs were first digested with 4 U/mL Elastase in DMEM/F12 for 15 min. Then tissues were chopped into small pieces, and digested further with 100 mg/mL DNase I in DMEM/F12 for 15 min. Digestion was terminated with DMEM/F12 medium. Single cells were filtered through 100 mm filter, spined down, and resuspend with 700 mL HBSS^+^ buffer for downstream experiments.

### 2.4. Flow Cytometry

Flow cytometrical analysis was described previously [6,13]. Mouse lung single cell suspension was stained with fixable viability dye, EPCAM, CD45, CD31, CD34, PDGFRa^+^, Sca-1 and CD24. AEC2s were gated as live EPCAM^+^CD45^−^CD31^−^CD34^−^Sca-1^−^CD24^−^ cells, PDGFRa^+^ fibroblasts were gated as live EPCAM^−^CD45^−^CD31^−^CD34^−^ PDGFRa^+^ cells. Primary antibodies Percp-Cy5.5-EPCAM (clone G8.8, catalog# 118220), APC-Cy7-CD45 (clone 30-F11, catalog# 103116), APC-Cy7-CD31 (clone MEC13.3, catalog# 102534), APC-Cy7-CD34 (clone HM34, catalog# 128622), FITC-CD24 (clone M1/69, catalog# 101806), PE-Cy7-Sca-1 (clone D7, catalog# 108114) and APC-PDGFRa^+^ (clone APA5, catalog# 135908) were from BioLegend (San Diego, CA, USA). Fixable viability dye (catalog# 65-0865-14) was from Thermo Fisher (Waltham, MA, USA). Stained cells were sorted on Aria III (BD Immunocytometry Systems; Franklin Lakes, NJ, USA) or analyzed on Fortessa (BD Immunocytometry Systems) and the data were analyzed with FlowJo software.

### 2.5. Cell Cycle Analysis

Surface marker-stained single cell suspension was fixed with Foxp3/Transcription Factor Staining Buffer Set (catalog# 00-5523-00, Thermo Fisher) for 1 h, then stained with conjugated APC-Ki-67 antibody (clone 16A8, catalog 652406, BioLegend) for another hour, then cells were washed, resuspended, and analyzed on Fortessa and the data were analyzed with FlowJo software.

### 2.6. RNA Isolation and Analysis

RNA isolation and analysis was described previously [6,24]. Briefly, 500 mL Trizol reagent (catalog# 15596026, Thermo Fisher) was added to freshly isolated PDGFRa^+^ fibroblasts, and incubated at room temperature for 5 min. 100 mL chloroform solution was added and incubated for 3 min. 250 mL isopropanol was then added and incubated for another 10 min. The mixture was then centrifuged for 10 min at 13,500 rpm, and supernatant was discarded, pellets were washed with 500 mL of 75% ethanol, followed by centrifuging for 5 min at 7500 *g* at 4 °C. After removal of the supernatant, RNA was air dried for 10 min, resuspend in 30 mL RNase-free water. Total RNAs were sent for RNA-Seq and reverse-transcribed into cDNA for real-time PCR.

### 2.7. Immunofluorescence

Mouse lung tissues were fixed with 10% formalin solution and embedded in paraffin. The tissues were sectioned, and the slices were blocked with 5% goat serum, stained with primary antibodies against pro-SPC (catalog# ab170699, Abcam; Boston, MA, USA) overnight at 4 °C. On the next day, the samples were washed with PBS+0.01% Tween 20 buffer and stained with secondary antibody. Results were visualized with Zeiss LSM 780 confocal microscope (Carl Zeiss AG, Oberkochen, Germany).

### 2.8. Hydroxyproline Assay

Hydroxyproline assay to determine collagen contents in mouse lungs was described previously [6,24]. In brief, lung tissues were heat-dried, minced, and hydrolyzed overnight. The other day, pH was adjusted and samples were diluted with PBS. Then, samples were incubated with Chloramine T solution, perchloric acid and P-DMAB solution. Finally, samples were read under 557 nm wavelength. The ability of the assay to completely hydrolyze and recover hydroxyproline from collagen was confirmed using samples containing known amounts of purified collagen.

### 2.9. 3D Organoid Cultures of AEC2s

AEC2 organoid assays were described previously [6,12,13,24,42]. Col1a2-Cre;*Arrb1*^fl/fl^ and *Arrb1*^fl/fl^ mice were treated with 1.25 U/kg bleomycin. 14 days after treatment, 1 × 10^5^ fresh isolated lung PDGFRa^+^ fibroblasts were mixed with 3000 flow-sorted WT AEC2 cells, resuspended in 100 μL matrigel/medium (1:1) mixture (catalog# 356252, Corning Inc.; Corning, NY, USA) and plated into 24-well transwell insert, and cultured for another 14 days. For colony-formation assay with CCL7, WT AEC2 cells were pre-treated with 10 ng/mL recombinant CCL7 protein (catalog# 250–08, Peprotech; Cranbury, NJ, USA) at 37 °C for 30 min, then mixed with mlg2908 fibroblasts. 400 μL medium was added to the lower chamber of the insert with a half medium change every other day. In the treatment group, 10 ng/mL CCL7 was also added to lower chamber medium. 14 days after culturing, colonies were counted and colony-formation efficiency was calculated.

### 2.10. Statistics

Results were shown as mean ± SD, statistical difference was calculated with Prism 8 (GraphPad Software, San Diego, CA, USA). Student’s two-tailed *t* test was used for two-group comparisons. Results were considered statistically significant at *p* < 0.05.

### 2.11. Data and Material Availability

The data used in this paper can be accessed via GSE47460 [43] and GSE48455 [44]. Raw data of RNA-seq is available upon request. Further information and requests for resources and reagents should be directed to D. Jiang (Dianhua.Jiang@cshs.org).

## 3. Results

### 3.1. Arrb1 Deficiency in Fibroblasts Protects Mice from Lung Fibrosis

**Figure 1.**
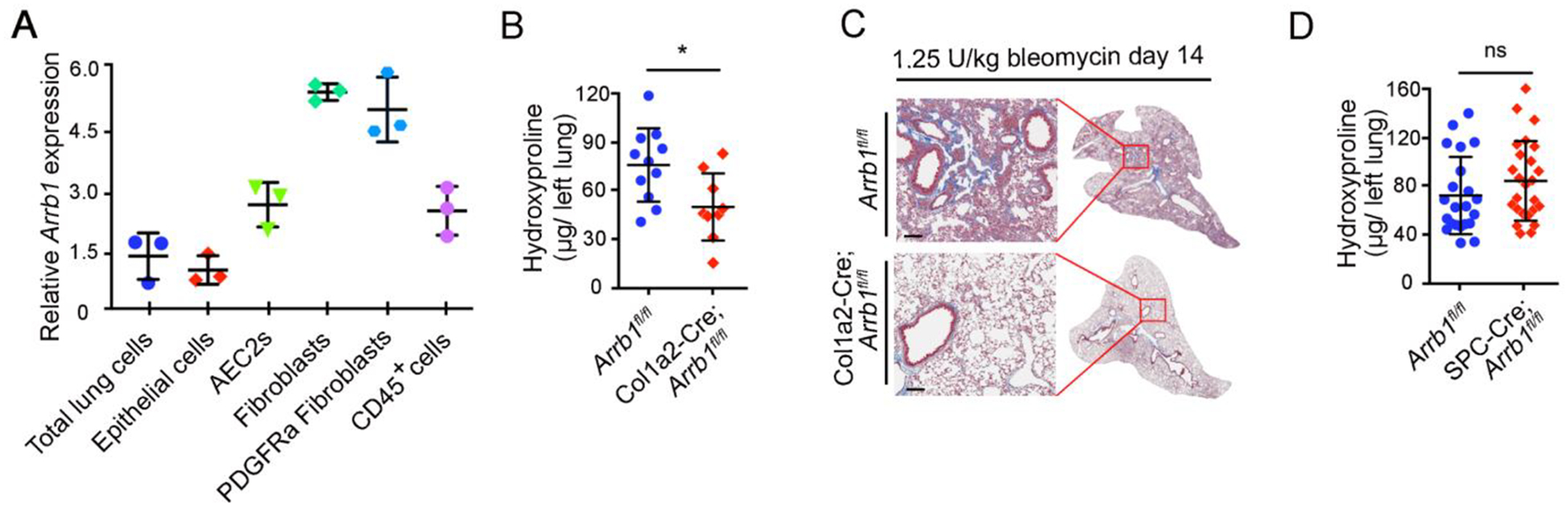
Arrb1 deficiency in fibroblasts protects mice from lung fibrosis. (**A**) Expression of *Arrb1* in different cell types from mouse lungs was determined. Cells were isolated via FACS, and total RNAs were extracted. *Arrb1* expression was determined with RT-PCR (*n* = 3). (**B**) Hydroxyproline assays of mouse left lungs 14 days after 1.25 U/kg bleomycin treatment (*Arrb1*^*f*l/fl^ *n* = 11, Col1a2-Cre; *Arrb1*^*f*l/fl^ *n* =9, *p < 0.05). (**C**) Trichrome staining of mouse lungs 14 days after 1.25 U/kg bleomycin injury. (**D**) Hydroxyproline assays of mouse left lungs 14 days after 1.25 U/kg bleomycin treatment (*Arrb1*^*f*l/fl^ *n* = 22, SPC-Cre; *Arrb1*^*f*l/fl^ *n* =25, *p < 0.05).

Our previous data showed that mice with conventional deletion of *Arrb1* were protected from bleomycin-induced injury and fibrosis [37]. To study the cell type responsible for this protective role, we analyzed *Arrb1* expression in different cell types in the lung. Results showed that *Arrb1* transcripts were highly expressed in lung fibroblasts, especially PDGFRa^+^ fibroblasts (Figure 1A). We further generated a mouse strain with lung fibroblast-specific deficiency of *Arrb1*. When challenged with bleomycin, mice with lung fibroblast-specific deficiency of *Arrb1* showed to a decrease in hydroxyproline content in the lungs compared to littermate control mice (Figure 1B), and reduced lung fibrosis was confirmed with Trichrome staining (Figure 1C). In contrast, deletion of Arrb1 in SPC^+^ AEC2 compartment did not affect fibrosis when challenged with bleomycin (Figure 1D). These data demonstrate a detrimental role of ARRB1 in fibroblasts in bleomycin-induced lung fibrosis.

### 3.2. Enhanced AEC2 Renewal in Mouse Lungs with Arrb1 Deficiency in Fibroblasts In Vivo

**Figure 2.**
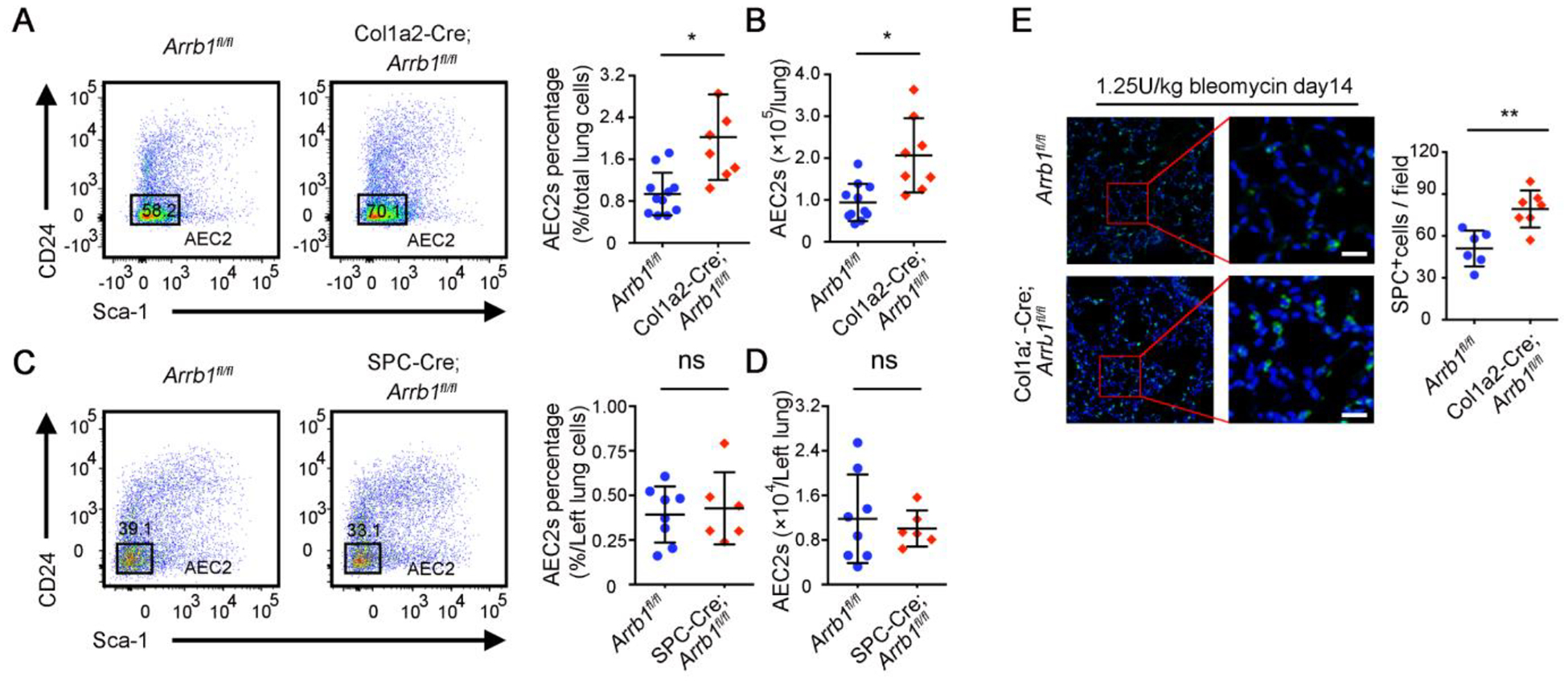
*Arrb1* deficiency in fibroblasts leads to increased AEC2 recovery in injured mouse lung. (**A**–**D**) 14 days after 1.25 U/kg bleomycin treatment, AEC2 cells were analyzed as live CD45^−^CD31^−^CD34^−^Epcam^+^CD24^−^Sca-1^−^ cells with flow cytometry. Percentage (**A**, *Arrb1*^*f*l/fl^ *n* = 11, Col1a2-Cre; *Arrb1*^*f*l/fl^ *n* = 7; *p < 0.05) and total number (**B**, *Arrb1*^*f*l/fl^ *n* = 11, Col1a2-Cre; *Arrb1*^*f*l/fl^ *n* = 8; *p < 0.05) of AEC2 cells in Col1a2-Cre;*Arrb1*^*f*l/fl^ mouse lungs as well as percentage (**C**, *Arrb1*^*f*l/fl^ *n* = 8, SPC-Cre;*Arrb1*^*f*l/fl^ *n* = 6; ns, not significant) and total number (**D**, *Arrb1*^*f*l/fl^ *n* = 8, SPC-Cre; *Arrb1*^*f*l/fl^ *n* = 6; ns, not significant) of AEC2 cells in SPC-Cre;*Arrb1*^fl/fl^ mouse lungs were calculated according to flow results. (**E**) Immunofluorescence staining of SPC^+^ cells from mouse lungs 14 days after 1.25 U/kg bleomycin treatment (*Arrb1*^*f*l/fl^ *n* = 7, Col1a2-Cre; *Arrb1*^*f*l/fl^ *n* = 8; **p < 0.01).

The mounting evidence indicates that the stem cell niche plays a crucial role in governing the regeneration of stem cells [45]. We recently showed that injured fibroblasts were less supportive role of AEC2 renewal [18]. We thus investigated the potential impact of *Arrb1* deletion in fibroblasts on the regeneration of AEC2. We found that mouse lungs with fibroblast-specific *Arrb1* deficiency have a higher percentage (Figure 2A) and number (Figure 2B) of AEC2 cells after bleomycin treatment. In contrast, deletion of *Arrb1* in SPC^+^ AEC2 compartment did not affect the percentage and number of AEC2 cells after bleomycin treatment (Figure 2C,D). These data indicated that deficiency of *Arrb1* in fibroblasts may promote AEC2 regeneration after injury. The results were further confirmed with immunofluorescence staining of pro-SPC (Figure 2E). These data showed that *Arrb1* deficiency in fibroblasts leads to enhanced recovery of AEC2 cells after lung injury.

### 3.3. Arrb1-deficient Fibroblasts Promotes AEC2 Proliferation and Renewal

**Figure 3.**
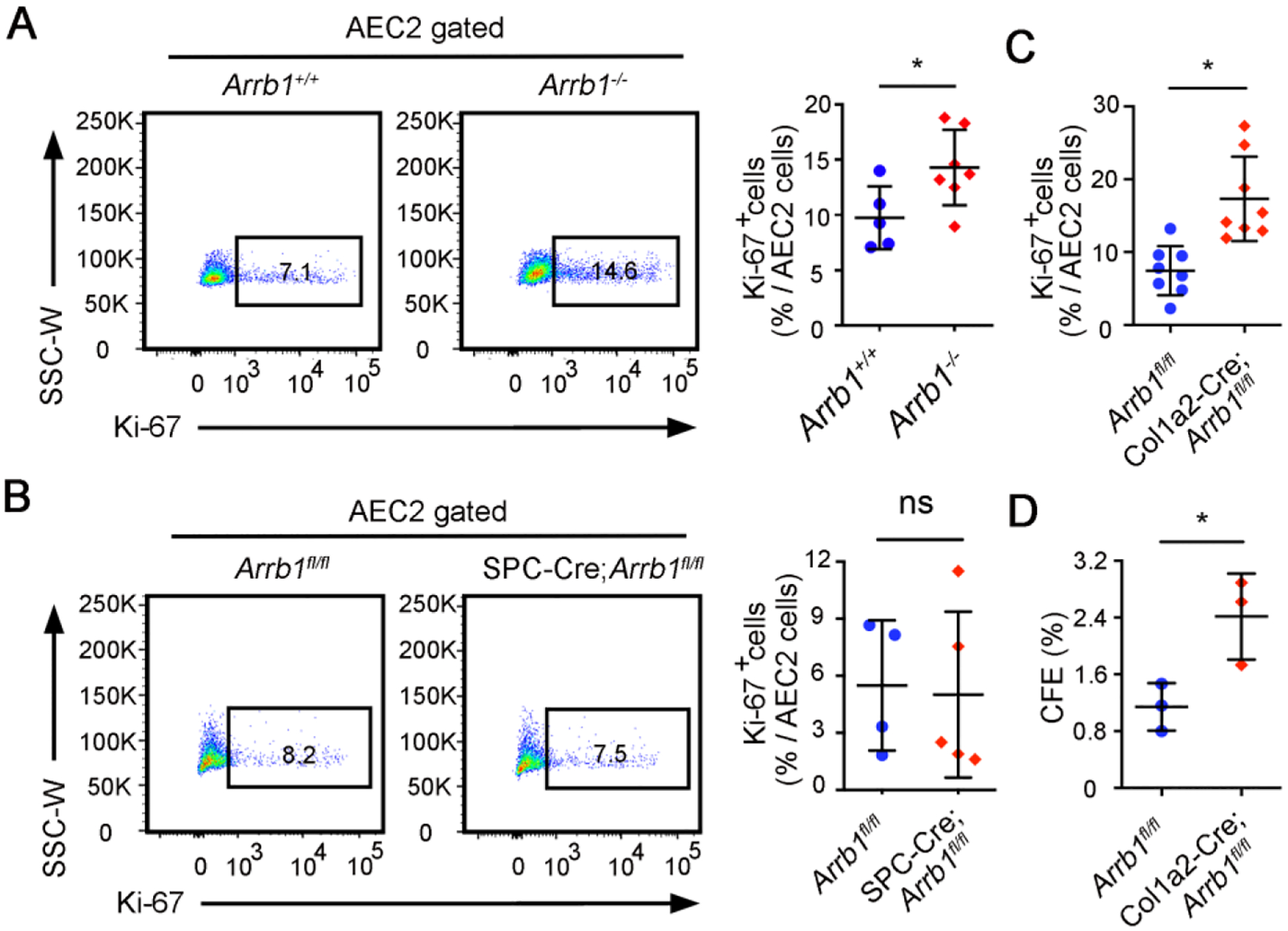
*Arrb1*-deficient fibroblasts promote AEC2 proliferation and renewal. (**A**–**C**) All mice were subjected to 1.25 U/kg bleomycin and mouse lungs were harvested 14 days after injury. AEC2 cells were gated as live CD45^−^CD31^−^CD34^−^Epcam^+^CD24^−^Sca-1^−^, percentage of Ki-67^+^ cells were calculated. Ki-67^+^ AEC2 cells were analyzed by FACS in *Arrb1*^+/+^ and *Arrb1*^−/−^ mice (**A**, *Arrb1*^+/+^ *n* = 5, *Arrb1*^−/−^ *n* = 7; *p < 0.05), *Arrb1*^fl/fl^ and SPC-Cre;*Arrb1*^fl/fl^ mice (**B**, *Arrb1*^fl/fl^ *n* = 7, SPC-Cre;*Arrb1*^fl/fl^ *n* = 6; ns, not significant), *Arrb1*^fl/fl^ and Col1a2-Cre; *Arrb1*^fl/fl^ mice (**C**, *n* = 8; *p < 0.05). (**D**) AEC2 cells from wild type C57BL/6 mice were cocultured with freshly isolated PDGFRa^+^ fibroblasts (CD45^−^CD31^−^CD34^−^EPCAM^−^PDGFRa^+^) from *Arrb1*^fl/fl^ and Col1a2-Cre;*Arrb1*^fl/fl^ mice lungs 7 days after 1.25 U/kg bleomycin treatment. 14 days after culture, colony-formation efficiency (CFE) was calculated (*n* = 3, *p < 0.05).

AEC2 cells are adult stem cells in the lung, and regenerate the alveolar epithelial cells after injury [12,13]. To further demonstrate that ARRB1 affects AEC2 regeneration, we first assessed AEC2 proliferation in *Arrb1*^−/−^ total knockout mice after bleomycin injury. The results showed that 7 days after bleomycin, there are more Ki-67^+^ AEC2 cells in *Arrb1*^−/−^ mice compared to control mice (Figure 3A). However, the increase of Ki-67^+^ AEC2 cells was not observed in the lungs of mice with *Arrb1* deletion specifically in AEC2s (SPC-Cre;*Arrb1*^fl/fl^ mice) (Figure 3B), indicating that ARRB1 promotes AEC2 proliferation via fibroblasts rather than AEC2s themselves. Indeed, when we deleted Arrb1 in Col1a2^+^ fibroblasts, an increase of Ki-67^+^ AEC2 cells was observed (Figure 3C). To further validate that *Arrb1*^−/−^ fibroblasts promote AEC2 proliferation, WT AEC2 cells were cocultured with *Arrb1*-deficient PDGFRa^+^ fibroblasts. Results showed that *Arrb1* deficiency in fibroblasts significantly increased colony-formation ability of AEC2 cells (Figure 3D), confirming that ARRB1 from fibroblasts affects AEC2 cell proliferation and renewal.

### 3.4. ARRB1 Promotes CCL7 Expression in Fibroblasts

**Figure 4.**
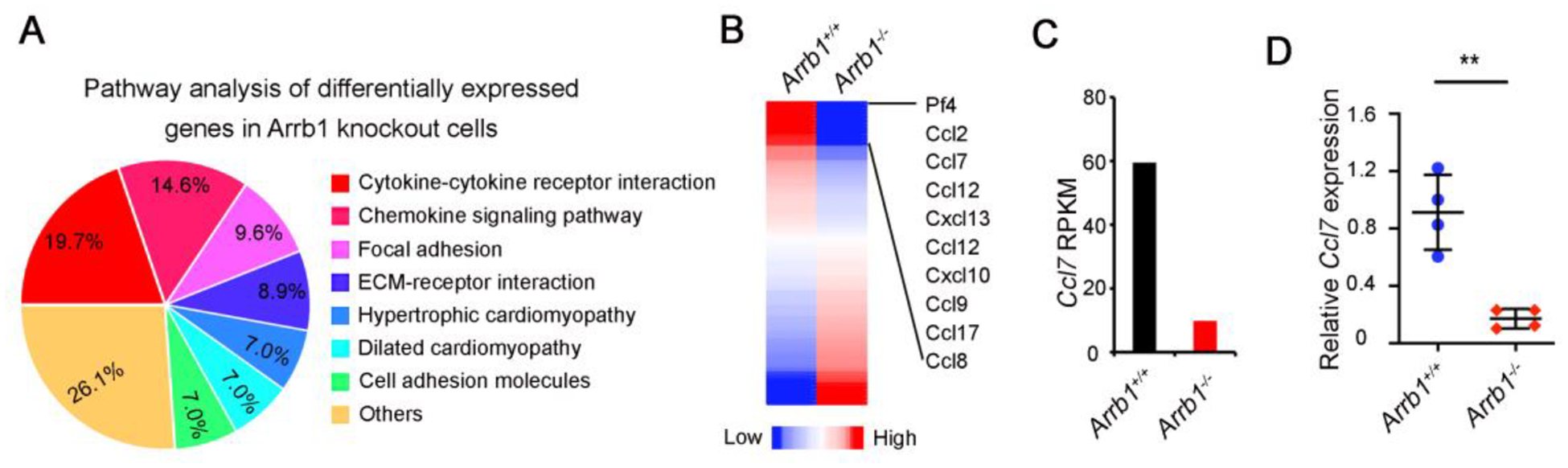
Arrb1 promotes Ccl7 expression in fibroblasts. (**A**) *Arrb1*^+/+^ and *Arrb1*^−/−^ mice were subjected to 1.25 U/kg bleomycin injury, PDGFRa^+^ fibroblasts were isolated with FACS 7 days after treatment. RNA-seq was performed with total RNAs isolated from these cells. Pathway analysis was conducted with Ingenuity Pathway Analysis (IPA) on the differentially expressed genes (DEGs). (**B**) Differentially expressed cytokines and chemokines were extracted. C–C chemokine genes are indicated. (**C**) RPKM of *Ccl7* in PDGFRa^+^ fibroblasts from *Arrb1*^+/+^ and *Arrb1*^−/−^ lungs in RNA-Seq analysis. (**D**) *Ccl7* expression in PDGFRa^+^ fibroblasts was analyzed with RT-PCR (*n* = 4; **p < 0.01).

Next, we sought to determine how *Arrb1*-deficent fibroblasts promote AEC2 proliferation. We isolated PDGFRa^+^ fibroblasts from *Arrb1*^+/+^ and *Arrb1*^−/−^ mice, and performed RNA sequencing (RNA-Seq). Results showed that differentially expressed genes were enriched in cytokine-cytokine receptor interactions and chemokine signaling pathways (Figure 4A). Cytokines and chemokines analysis showed that *Ccl7* was among the top differential expressed genes in *Arrb1* deficiency (Figure 4B). RPKM results also confirmed that *Ccl7* was downregulated in *Arrb1*-deficient fibroblasts (Figure 4C). We confirmed these results by RT-PCR analysis, showing that Ccl7 expression was decreased in *Arrb1*-deficient fibroblasts (Figure 4D). Thus, these data demonstrated that *Arrb1* deficiency downregulates *Ccl7* expression in fibroblasts.

### 3.5. CCL7 Inhibits AEC2 Renewal In Vitro

Our data show that mice with *Arrb1* deficiency in fibroblasts were protected from bleomycin-induced lung fibrosis, *Arrb1*-deficient fibroblasts promoted AEC2 proliferation, and *Arrb1* deficiency in fibroblasts led to decreased Ccl7 expression. CCL7 (C–C Motif Chemokine Ligand 7, aka Monocyte Chemoattractant Protein 3, MCP3) is a member of the C–C subfamily of chemokines [46], which plays a role in macrophage recruitment during inflammation [47] and in tumor metastasis [46]. *Ccl7* is among several upregulated inflammatory genes identified during early injury [44].

**Figure 5.**
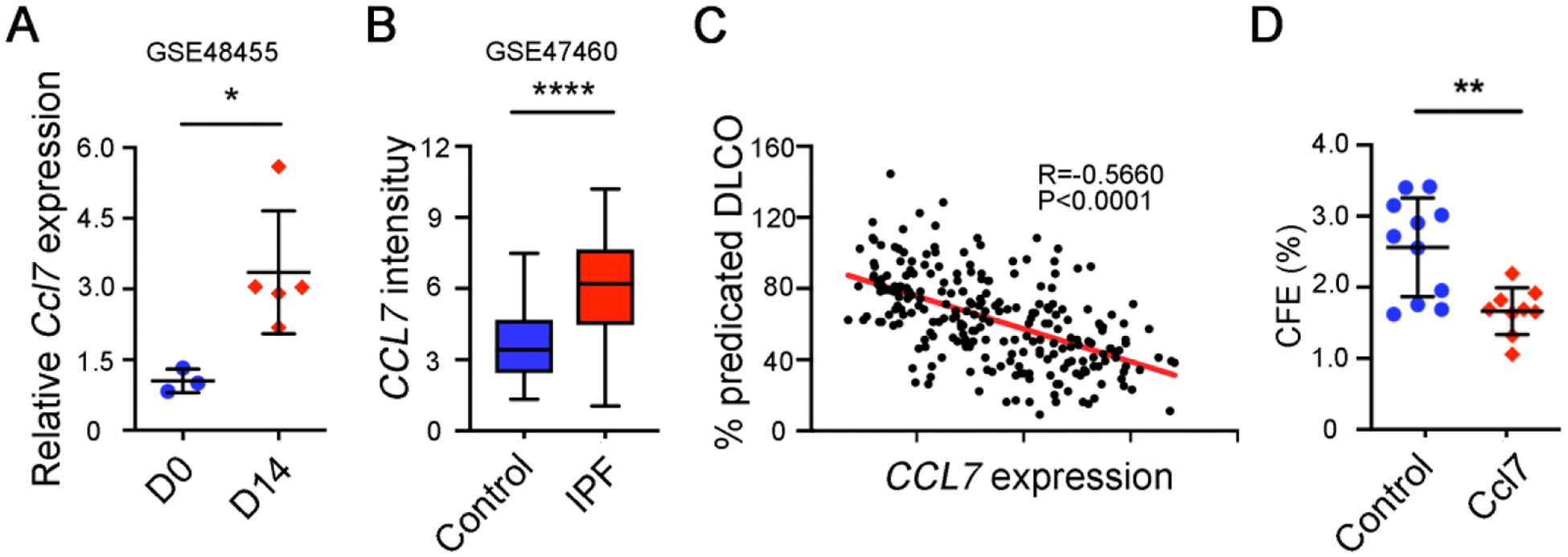
CCL7 inhibits AEC2 renewal. (**A**) *Ccl7* expression on day 0 and day 14 rat lungs after bleomycin treatment in GSE48455 dataset was analyzed (Day 0 *n* = 3, Day 14 *n* = 5, *p < 0.05). (**B**) *CCL7* expression in IPF patients and healthy controls in GSE47460 dataset was analyzed (Control *n* = 108, IPF *n* = 160, ****p < 0.0001). (**C**) Pearson correlation of *CCL7* expression and the percentage of predicted diffusion capacity for carbon monoxide (DLCO) in GSE47460 dataset was determined (*n* = 242, *R* = −0.5660, *p* < 0.0001). (**D**) Colony formation efficiency of AEC2 from wild type C57BL/6 mice treated with 10 ng/mL CCL7 (Control *n* = 11, CCL7 *n* = 9; **p < 0.01).

Next, we determined whether CCL7 directly affects AEC2 renewal. Analysis of rat lung injury data [44] showed that Ccl7 expression was increased after bleomycin treatment (Figure 5A). Importantly, CCL7 expression was also found to be increased in lungs of IPF patients compared to healthy lungs (Figure 5B). Furthermore, CCL7 expression is correlated with reduced lung function shown as the negative correlation between CCL7 expression and the percentage of predicted diffusion capacity for carbon monoxide (DLCO) (Figure 5C). In addition, 3D organoid experiments showed that adding CCL7 in organoid assays significantly decreased AEC2 renewal capacity (Figure 5D), confirming that CCL7 inhibits AEC2 regeneration.

In summary, these results demonstrate that fibroblast ARRB1 mediates AEC2 regeneration, possibly by releasing chemokine CCL7, which inhibits AEC2 regeneration, leading to fibrosis in the lung.

## 4. Discussion

AEC2s are adult stem cells in the lung [12]. We and others have demonstrated a loss of AEC2s and a failure of AEC2 renewal in IPF lungs [13,14]. We previously reported that beta-arrestins are the key regulators of fibroblast invasion, and beta-arrestin 1 and 2 deficient mice exhibit decreased lung fibrosis [37]. However, the role of beta-arrestins in AEC2 regeneration is unclear. In this study, we investigated the role of ARRB1 in AEC2 renewal and in lung fibrosis and found that *Arrb1* deficiency in fibroblasts enhanced AEC2 regeneration and protected mice from lung fibrosis.

AEC2 stem cell regenerative capacity is regulated by the AEC2 stem cell niche [18,21–23]. We recently showed that basal-like cells may produce WNT ligands, mediating AEC2 renewal [24]. Several studies have showed that lipofibroblasts and PDGFRa^+^ fibroblasts regulate alveolar progenitor cell activities [18,25,26]. Other cell types such as endothelial cells [20,27,28], macrophages [17,29], and T cells [30] regulate alveolar progenitor cell activities. The current study is consistent with these studies and makes the novel observation that fibroblast ARRB1 is crucial for alveolar progenitor cell regeneration. Beta-Arrestins are known to act as a scaffold to regulate GPCR signaling through receptor desensitization and internalization [31–33]. Further studies are needed to determine how ARRB1 interacts with specific GPCRs in fibroblasts for this effector function.

Chemokine CCL7 (MCP3) is a member of the C–C subfamily [46]. It has been shown to play a role in macrophage recruitment during inflammation [47]. Both lung macrophages and myofibroblasts secrete CCL7 into the blood [48]. We found that CCL7 is increased in IPF and negatively correlated with lung function, consistent with a previous report showing an increase in CCL7 in fibroblasts from patients with usual interstitial pneumonia [49], in serum of patients with systemic sclerosis [50], and a recent preprint showing a significant increase of CCL7 in IPF plasma compared to that of matched controls [48]. We showed that CCL7 is also increased after bleomycin injury in mice, consistent with a previous report that CCL7 and CCL2 (MCP1) are increased in the BALF of Sftpc BRICHOS mutant mice [51]. CCL7 may also be a feature of aging [52]. Inhibition of CCL7 receptor CCR2 enhanced aged muscle regeneration and functional recovery after skeletal muscle injury [52].

CCL7 is known for its role in macrophage recruitment, mediating immune response [46]. It is reported that CCL7 promotes activation of the TGF-beta signaling pathways leading to increased type I collagen production [53]. We previously reported that beta-arrestins are key regulators of fibroblast invasion [37]. It is unclear if CCL7 has a role in fibroblast invasion. In this study, we showed that fibroblast-derived CCL7 participates in the AEC2 stem cell niche and inhibits AEC2 renewal. We previously showed that CXC chemokines such as CXCL1 [18] and cytokines such as IL-6 promote AEC2 renewal [13,18]. CCL7 binds CCR2 [54], CCR1, and CCR3, which are typical GPCRs. Future studies are needed to determine which and how CCL7 receptor participates in the AEC2 niche. At the cellular level, CCL7 may bind to CCR2 on macrophages, the latter may negatively regulate the AEC2 niche. Macrophages can influence lung stem cells in a pneumonectomy model in mice [29]. Since CCR2 expression level on AEC2 cells is notably scant, it is unlikely that CCL7 inhibits AEC2 regeneration through CCL7–CCR2 interactions on AEC2s. Future studies are warranted to determine if the effect of CCL7 on AEC2 renewal is direct or indirect.

In conclusion, we demonstrate that fibroblast ARRB1 impedes AEC2 regeneration, possibly by releasing chemokine CCL7, leading to fibrosis in the lung.
